# Education, Smoking and *CRP* Genetics in Relation to C-Reactive Protein Concentrations in Black South Africans

**DOI:** 10.3390/ijerph17186646

**Published:** 2020-09-11

**Authors:** Pieter Hermanus Myburgh, Cornelie Nienaber-Rousseau, Iolanthé Marike Kruger, Gordon Wayne Towers

**Affiliations:** 1 Institute for Intelligent Systems, University of Johannesburg, Johannesburg 2006, South Africa; hermanm@uj.ac.za; 2 Centre of Excellence for Nutrition, Faculty of Health Sciences, North-West University, Potchefstroom 2520, South Africa; 3 Medical Research Council Unit for Hypertension and Cardiovascular Disease, North-West University, Potchefstroom 2520, South Africa; 4 Africa Unit for Transdisciplinary Health Research, Faculty of Health Sciences, North-West University, Potchefstroom 2520, South Africa; lanthe.kruger@nwu.ac.za (I.M.K.); wayne.towers@nwu.ac.za (G.W.T.)

**Keywords:** CVD, inflammation, schooling, SES, smoking, socio-economic status

## Abstract

Because elevated circulating C-reactive protein (CRP) and low socio-economic status (SES), have both been implicated in cardiovascular disease development, we investigated whether SES factors associate with and interact with *CRP* polymorphisms in relation to the phenotype. Included in the study were 1569 black South Africans for whom CRP concentrations, 12 *CRP* single nucleotide polymorphisms (SNPs), cardiovascular health markers, and SES factors were known. None of the investigated SES aspects was found to associate with CRP concentrations when measured individually; however, in adjusted analyses, attaining twelve or more years of formal education resulted in a hypothetically predicted 18.9% lower CRP concentration. We also present the first evidence that active smokers with a C-allele at rs3093068 are at an increased risk of presenting with elevated CRP concentrations. Apart from education level, most SES factors on their own are not associated with the elevated CRP phenotype observed in black South Africans. However, these factors may collectively with other environmental, genetic, and behavioral aspects such as smoking, contribute to the elevated inflammation levels observed in this population. The gene-smoking status interaction in relation to inflammation observed here is of interest and if replicated could be used in at-risk individuals to serve as an additional motivation to quit.

## 1. Introduction

Non-communicable diseases (NCDs) accounted for 71.3% of global deaths between 2005 and 2015 [1]. Of these NCDs, cardiovascular disease (CVD) took the highest toll in developing nations such as South Africa [2]. Several CVDs share an inflammatory origin, which is influenced by numerous factors, including anthropometry, level of physical activity, and the genetic background of an individual [3]. One such marker of inflammation, which has been determined to predict future CVD risk, is the cytokine, C-reactive protein (CRP). Elevated levels of this protein, i.e., >3 mg/L, are predictive of future CVD [4,5]. CRP is generally elevated in black individuals, coinciding with notably stronger inflammatory responses as well as higher CVD risk than in other ethnicities [6,7,8,9]. Other factors besides ethnicity, such as age and gender, are also determinants of this cytokine, with indications that differing CRP concentrations could additionally be attributed to socio-economic status (SES) tiers [10].

SES is multi-layered, reflecting educational-employment factors, access to infrastructure (providing water, sanitation and electricity, for example) and quality of housing. SES affects the inflammatory state [11] as well as the CVD risk of an individual [12]. Various studies reported that lower SES co-presented with elevated CRP concentrations [11,13], while low SES in childhood was reported by a recent meta-analysis to result in 25% higher CRP concentrations during adulthood [10]. Recently, Egbujie et al. [14] linked SES to CVD risk in black South Africans, while another report indicated that the higher risk among black Americans of developing CVD could be offset by improved SES [15]. In South Africa, it was reported that a change in CVD risk factors was observed between 1996 and 2005, with CVD risk shifting from individuals with a high SES to those with lower SES [16].

The mechanisms by which SES affects CRP concentrations are still being debated, with different hypotheses currently under investigation. Lower SES is usually associated with poor diet, low levels of physical activity and higher incidence of tobacco and alcohol use [13]. Kershaw et al. [13] report that poverty and low levels of education were the main determinants of elevated CRP concentrations, with 87.9% of CRP variation attributed to education level being primarily explained by an increased prevalence of smoking, lower dietary quality and reduced levels of exercise.

Specific phenotypes, such as elevated CRP, are influenced not only by environmental factors, but also by clinical and genetic factors. Data from twin and family studies attribute up to 40% of heritability in CRP concentrations to heritable factors such as genetics; however, little is known about the genetics underpinning CRP in African populations [17,18]. Expression of a gene can be modulated by the environment and behavior of an individual, which may alter the phenotype observed [19]. To our knowledge, nothing has been reported on how different *CRP* single nucleotide polymorphisms (SNPs) interact with markers of SES and how this interaction could modulate CRP concentrations. We, therefore, investigated whether specific socio-economic factors associate with CRP concentrations, as a proxy for inflammation, and whether these factors mitigated the associations between specific *CRP* polymorphisms known to be associated with alterations in CRP concentrations. The interplay between SES, inflammation and the etiology of CVD could, therefore, be used to mitigate CVD risk by reducing inflammation through improving SES factors. Knowledge regarding the impact of SES on the genetic expression of CRP concentrations may help direct tailored preventive efforts for diseases contingent on inflammation.

## 2. Materials and Methods

This cross-sectional, observational study was nested within the South African arm of the Prospective Urban and Rural Epidemiology (PURE) study, with details of the sampling strategy described by Pisa et al. [16]. In total, 2010 apparently healthy adults (>30 years), from both rural and urban communities, were included at the baseline in 2005. Individuals with a measured fever (tympanic temperature > 38.0 °C), were excluded. Further exclusions were that participants could not have known acute overt pre-existing diseases, be pregnant or lactating at the time of sampling.

### 2.1. Biochemical Measurements

Fasting blood samples were collected by registered nurses. High-sensitivity CRP concentrations were measured on a Sequential Multiple Analyzer Computer (SMAC), using a particle-enhanced immunoturbidometric assay (Konelab™ autoanalyzer, Thermo Fisher Scientific Oy, Vantaa, Finland). Quantitative determination of high-density lipoprotein cholesterol (HDL-c), triglycerides and total cholesterol in the sera of participants were done on a Konelab™ 20i autoanalyzer (Thermo Fisher Scientific). Low-density lipoprotein concentrations (LDL-c) were calculated using the Friedewald equation for those with triglycerides below 400 mg/dL. Nurses trained in voluntary counseling and human immune deficiency virus (HIV) testing performed HIV tests in accordance with prevailing governmental and WHO guidelines. Pre-test counseling was provided in group format, after which signed informed consent was obtained individually. Those testing positive for HIV on a rapid First Response HIV1-2.O card test (Transnational Technologies Inc. PMC Medical, Nani Daman, India), were retested using a card test developed by Pareeshak (BHAT Bio-tech, Bangalore, India) to ensure diagnostic accuracy. All participants, irrespective of HIV status, received individual post-test counseling. Whole EDTA blood was used for measuring glycated hemoglobin (HbA1c) from fasting participants, with a D-10 Hemoglobin testing system (Bio-Rad Laboratories, Hercules, CA, USA).

### 2.2. Anthropometric and Physiological Measurements and Lifestyle Questionnaires

The participant’s body weight was measured in minimal clothing with arms hanging freely at the side. Weight was measured in duplicate, with the mean recorded. Height was measured in duplicate with a stadiometer, with the head in the Frankfort plane in a fully erect state while the participant inhaled. The mean was then calculated and recorded in meters. Body mass index (BMI) was calculated using the standard formula and reported as kg/m^2^. Waist circumference (WC) and hip circumference were measured using unstretchable metal tape in accordance with the recommendations of the International Society for the Advancement of Kinanthropometry. An Omron automatic digital blood pressure monitor (Omron HEM-757, Kyoto, Japan) was used to measure the right brachial artery blood pressure in the sitting position. Participants did not smoke, exercise, or eat 30 min beforehand, and had to be rested and calm for five minutes before measurement.

Volunteers responded to an interviewer-administered questionnaire in their language of choice, in which various socio-demographic variables (age, gender, medical history (stroke and diabetes incidence), tobacco use, alcohol usage, and SES factors (i.e., roof type, access to electricity, primary cooking fuel, primary heat source, water source, and education)) were collected. Water sources were grouped into sourced water (i.e., from wells, rivers, or boreholes) or municipal water sources. Even though we did not have access to a standardized SES-index, we overcame this by focusing on individual factors that constitute an individual’s living environment, to identify factors for which mitigation efforts could be instituted in an attempt to lower CRP concentrations. Food portion books were specifically designed and standardized for the South African PURE-North West population.

Validated, interviewer-based quantitative food frequency questionnaires or qFFQs [20] were completed to determine dietary intakes. The data obtained from qFFQs were entered into the Foodfinder3 program (Medical Research Council, Tygerberg, South Africa, 2007) and sent to the Medical Research Council of South Africa for nutrient analyses.

### 2.3. Genetic Analyses

Polymorphic sites and novel SNPs within the *CRP* gene were identified by sequencing in 30 randomly selected DNA samples and an in silico search. These variants were scored (varying from 0–1) by the Assay Design Tool to determine a viable customized genotyping array to be analyzed using the Illumina^®^ VeraCode GoldenGate assay technology on a BeadXpress^®^ platform (Illumina^®^ Inc., San Diego, CA, USA) for genotyping the selected SNPs. Ultimately only 12 *CRP* SNP clusters passed the quality control (QC) measures for making genotype calls by having a GenCall score >0.5 and a call rate ≥0.9 and are reported on here (see Table S1 for SNP details). The BeadXpress^®^ analysis was performed by the National Health Laboratory Service (NHLS) at the University of the Witwatersrand, Johannesburg.

### 2.4. Statistical Analyses

A total of 1569 individuals, for whom we had both CRP concentrations and all the genetic information regarding the SNPs investigated in the *CRP* gene, were included in our analyses. Statistical analyses were conducted using R [21]. Continuous variables were inspected for normality using histograms and measures of skewness. Variables with a skewed distribution were natural log-transformed and reported as median and interquartile ranges. Based on global recommendations for CRP cut-off values, data subsets were created i.e., ≤3 mg/L; >3 mg/L [5]. The compareGroups library was used to construct bivariate tables comparing our constructed cohorts, using non-parametric methods for both continuous and categorical data. Spearman correlations were computed, testing for linear associations with of continuous values, while median values and interquartile ranges for each categorical variable were reported. Significance testing was conducted using the independent two-group Mann–Whitney U test or the Kruskal–Wallis One-Way ANOVA by Ranks Test. A backward stepwise linear regression was conducted using the stepAIC function within the MASS library. Models were evaluated based on the Akaike information criterion (AIC) obtained. The final variables obtained were evaluated for co-linearity. Association analyses for SNP X environment interaction were then performed, using the SNPSassoc library, including the co-variates obtained from the linear regression model, and included based on lowest scoring AIC value. This was done for each SNP in combination with each demographic and SES factor. Where applicable, p-values were adjusted using the methods suggested by Bonferroni.

### 2.5. Ethics Statement

The authors and study coordinators complied with all ethical standards. The PURE-SA (North West province) study was approved by the Health Research Ethics Committee of the Faculty of Health Sciences, North-West University (NWU), in accordance with the ethical principles outlined by the Declaration of Helsinki with approval numbers 04M10, for the larger study, and NWU-00004-17-S1 for our affiliated study. Goodwill permission was granted by household heads and community leaders (mayors and traditional leaders), as well as the Department of Health of South Africa. Signed informed consent was given by each participant after being apprised of the aims of the study. Sufficient time for reflection was given, and subjects could withdraw at any time, or withhold whatever information they were not willing to share, without reprisal.

## 3. Results

### 3.1. Demographics and Anthropometrics of the Study Population and Their CVD-Risk Factors Stratified to at-Risk CRP Phenotypes

Women were more likely to present with elevated CRP concentrations (median unadjusted value of 3.58 mg/L). Post-menopausal women (self-reported with amenorrhea), had higher median CRP concentrations (4.31 [1.72; 11.9] mg/L) than men (2.42 [0.72; 7.87] mg/L) and pre-menopausal women (3.05 [0.82; 9.00] mg/L; *p* < 0.0001). Individuals with elevated CRP concentrations were physically larger than those with normal CRP, as indicated by higher BMI and other anthropometric markers, even though similar daily dietary intakes were noted. Post-menopausal women also had significantly larger WC (median: 82.4 cm) than pre-menopausal women (median: 79.0 cm) and men (median: 74.2 cm). After adjusting for WC, which differed between the genders (*p* < 0.0001), the difference in CRP concentrations observed between men and women, as well as pre- and post-menopausal women, disappeared. Those with elevated CRP were also significantly older, although age was only weakly, but significantly, associated with CRP (ρ = 0.12). Median CRP concentrations were similar irrespective of HIV status, tobacco and alcohol use. Smokers had a lower median WC (74 cm) as opposed to grouped individuals who had never smoked or were former smokers (81.4 cm, *p* < 10^−12^).

Median CRP concentrations were similar (*p* > 0.05) in rural and urban participants (Table 1), with similar proportions of individuals being classified as having normal or elevated CRP concentrations observed in these two areas. Factors pertaining to SES differed between the two localities (data not shown). Rural participants were more likely to be married and have lower education levels than urbanites, thereby pointing toward a lower SES level for rural dwellers. Ruralists were also more likely to access public water systems such as communal wells, to use wood as a primary heating and cooking fuel source, and have roofs constructed of corrugated iron sheeting with no insulation.

Next, we stratified factors pertaining to SES according to CRP risk values (Table 2). Except for marital status, similar distributions and median CRP concentrations were observed for all investigated SES factors. Individuals presenting with normal CRP concentrations were more likely to identify as never being married; however, when adjusting for age and WC, similar CRP concentrations were observed across all marital status categories. Smokers had significantly lower formal educational attainment than non-smokers (data not shown).

Individuals with elevated CRP concentrations presented with significantly poorer markers of CVD risk than those with normal CRP concentrations (Table 3). Cases of elevated CRP were prone to co-present with increased blood pressure, increased heart rate and a poorer lipid profile. Median glycated hemoglobin concentrations were also increased in individuals with elevated CRP concentrations.

To describe CRP concentrations and the interactions of modulators thereof on a physiological scale, natural log-transformed CRP (lnCRP) concentrations were modeled using a stepwise, backward linear regression approach. Eight statistically significant predictors were identified from the measured variables, including clinical, demographic and socio-economic factors (Table 3 and Table 4). The model presented accounted for 14.3% of the variation observed in CRP concentrations of our black population. A 22.0% predicted reduction in CRP concentration was observed in response to an increase of 1 mmol/L in HDL-c. All SES elements investigated in this study failed to predict CRP concentrations, except for whether an individual had attained 12 or more years of formal education, which resulted in a predicted reduction of 18.9% in CRP concentrations.

### 3.2. Effects of SES Factors on Association between Different CRP Genotypes and CRP Concentrations

**Figure 1:**
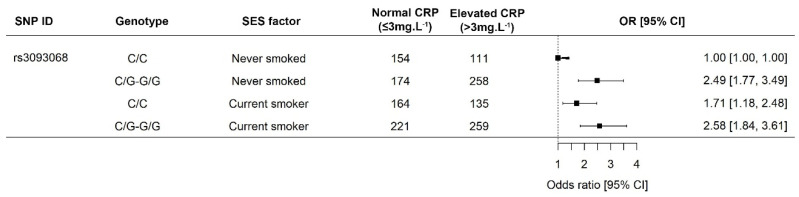
Interaction between tobacco smoke and rs3093068 in the Prospective Urban and Rural Epidemiology study—North West arm. The minor allele is associated with increased CRP concentrations, which are further increased in smokers. Men were more likely to be current smokers (59.5% vs. 47.6%; *p* < 0.0001). Homozygous smokers for the minor allele had a 71% increased risk of presenting with elevated CRP concentrations. Abbreviations: CRP, C-reactive protein; C, cytosine; CI, 95% confidence interval; G, guanine.

The odds of presenting with elevated CRP concentrations were independently investigated for each demographic or SES component included in this study in combination with each of the twelve *CRP* genotypes. The only significant interaction observed in our population was that of smoking status in individuals of differing rs3093068 genotypes. Individuals indicating that they were former smokers were included in our association analysis as abstainers to enable sufficient statistical power. Smokers had lower median WC (74 cm) as opposed to individuals who had never smoked or were former smokers (81.4 cm, *p* < 10^−12^). In contrast, current smokers presented with the higher median daily dietary intake (7306 kJ) than current non-smokers (7037 kJ). The odds of presenting with elevated CRP concentrations were 71% higher for those homozygous for the minor allele (C/C) than non-smokers (Figure 1). Individuals with the wild-type had similar odds of presenting with elevated CRP concentrations, irrespective of their smoking status.

## 4. Discussion

Little evidence exists on whether—and indeed, if—individual SES factors that constitute an individual’s immediate living environment affect their inflammatory status. In this study, we failed to find sufficient evidence that the investigated SES elements acted individually as impetus for elevated CRP concentrations, the exception being that lower CRP concentrations were predicted from adjusted analyses in individuals completing at least 12 years of formal education. Our evidence, however, highlights the fact that the inflammatory phenotype observed in black populations is the result of a combination of various factors, including, but not limited to, the combined effects of genetics with individual lifestyle choices such as smoking. Moreover, our results indicated that black participants with CRP concentrations above 3 mg/L have a higher prevalence of CVD risk factors.

Several epidemiological studies exclude individuals with CRP concentrations above 10 mg/L, which is seen as the clinical cut-off point for acute infections. However, [22] reported that certain individuals, especially obese women, had repeatedly presented with CRP concentrations above 10 mg/L without any indication of acute infection. In our study, all individuals examined had normal body temperatures, reducing the likelihood of acute infection as a cause of excessively elevated CRP concentrations in the 363 (23.1%) individuals presenting with CRP concentrations above 10 mg/L. Nienaber-Rousseau et al. (unpublished) proved statistically that excluding participants within our population with CRP concentrations higher than 10 mg/L leads to the exclusion of certain CRP genotypes, which results in a biased representation of the actual drivers of increased CRP concentrations observed in black African populations. Furthermore, we included these individuals as excluding them would have decreased the statistical power when stratifying within the different SES components and different genotypes. We also included individuals who were seropositive for HIV, as median CRP values were similar regardless of HIV status. Infection rates are also higher among individuals with low SES, which could result in the introduction of bias should HIV-positive individuals have been excluded [23].

Elevated CRP concentrations were regularly observed in the women included in our study. Study [24] reported that black women were more likely to have CRP concentrations above 3 mg/L and that elevated CRP was more frequently observed in post-menopausal women, although it was strongly correlated with abdominal obesity. Likewise, gender as well as pre-menopausal and post-menopausal differences dissipated when we corrected for WC in our study, implicating WC as a major contributing factor to the development of an elevated CRP phenotype. Anthropometric markers such as waist and hip circumferences, weight and BMI had significant positive correlations with CRP concentration (Table 2, ρ = 0.27, 0.21, 0.22 and 0.24, respectively). Various other reports record the influence of adiposity on the inflammatory state of the individual [25,26]. The association between BMI and CRP, irrespective of ethnicity, was reported in another study [27], and elevated CRP concentrations, as well as increased CVD risk, are often the result of increased adiposity [26].

Using CRP as a prognostic marker for future CVD risk appears to be independent of ethnic or geographical factors [6]. Factors pertaining to CVD risk were observed as being elevated in individuals harboring elevated CRP concentrations in our sample. Similar to our findings, a multi-ethnic study reports increased resting heart rate to be associated with increased concentrations of inflammatory markers, including CRP [28]. Inflammation markers, and especially CRP, are also linked to vascular stiffness, atherosclerosis and the development of end-organ damage, characteristics of a long-term hypertensive state combined with hyperlipidemia [29]. African Americans are also reported to be more likely to exhibit elevated HbA1c concentrations, with CRP highly correlated with HbA1c levels [30]. Excessive weight, hyperlipoproteinemia, and decreased insulin sensitivity are traits associated with the metabolic syndrome or MetS [31]. Combined with the elevated inflammation levels, MetS was, therefore, prominent in the group of volunteers studied and even more so in post-menopausal women, regardless of their SES.

SES factors differed between urban and rural participants; however, CRP concentrations were similar regardless of where individuals resided. The lack of any impact exerted on CRP concentrations by SES elements (Table 2) further strengthens our observation that individual SES components are not the main causative effect of elevated CRP concentrations in this population. The detected similarity in CRP concentrations between different levels of urbanization with varying markers of SES is in contrast to observations made in an Asian population, where city dwellers had higher CRP concentrations [32]. The years following the fall of apartheid in South Africa were marked by unprecedented rates of urbanization, which improved economic activity and increased rural-to-urban migrations [16]. Furthermore, improved access to basic utilities resulted from governmental efforts, even in the rural areas included in this study [33]. It may, therefore, be argued that the definition of what constitutes a rural area differed between our two studies, which may have resulted in this discrepancy. Of all the included SES factors, only education was determined to be an influencer of CRP concentration, and only when controlling for other confounding variables.

Although some of the values in Table 4 suggest substantial changes in CRP for a single unit change in a specific variable, the interpretation should consider the physiological changes of such alterations. Age-dependent increases in CRP were associated with elevated adiposity due to changes in hormonal balances, as reported in previous studies [34] similar to our investigation. Substantial reductions in CRP were predicted with a 1 mmol/L change in HDL-c; however, eliciting this response may prove difficult in a resource-poor environment. These covariates, however, do predict possible routes of intervention, whereby proper nutrition (focusing on weight management, treatment of hyperlipidemia, and glycemic control), as well as increased physical activity (to improve resting heart rate) and increasing education levels, can reduce inflammation in populations [35,36,37]. Completing 12 or more years of formal education was associated with reduced CRP concentrations (Table 1, unadjusted), although this reduction was found to be non-significant. In our multivariate model, completing secondary school or tertiary education corresponded to a significant 18.9% reduction in predicted CRP concentration. The authors of [13] estimate that 87.9% of CRP variation attributed to education level could be primarily explained by the higher number of smokers, the lower dietary quality and reduced levels of exercise in lower educated individuals. Similarly, it was reported for our cohort that education levels were associated with lower BMIs in both men and women [16].

Various other studies have also failed to find differences in the CRP concentrations of smokers versus non-smokers, although smoking is known to affect CVD risk [38,39]. Smokers in our study had lower WC, with higher daily dietary intakes than non-smokers. Previously, African American smokers were reported to have lower levels of weight gain than white Americans [40]. However, nicotine does increase energy expenditure [40], which may have resulted in the smaller WC observed in active tobacco users in our study. To our knowledge, we present the first indication that smoking status results in increased CRP concentrations in individuals harboring the minor allele of rs3093068, of which the major allele is associated with increased CRP concentrations [19]. Smokers with the minor allele had odds of presenting with elevated CRP concentrations statistically similar to those with the wild-type, negating the CRP-lowering effects of the minor allele.

## 5. Conclusions

Our main findings suggest that CRP concentrations in black South Africans are not associated with individual SES factors. Even though the SES factors included are not primarily responsible for the elevated CRP concentrations observed, improving the general SES of individuals commonly results in better health outcomes. Therefore, there should be collective efforts to improve the general socio-economic standing of the people of the Republic of South Africa. Health promotion efforts should focus on reducing the individual symptoms that constitute MetS, with public health promotion efforts especially focused on individuals with lower education levels. Here we also presented the first evidence that smoking status increases CRP concentrations in individuals who are homozygous for the minor allele of rs3093068, although more evidence is needed from other ethnicities. Our data were also cross-sectional in nature, and, therefore, do not account for changes in SES factors for which future elevated CRP concentrations were yet to be moderated by improvements in these SES factors. Future studies measuring SES factors should, consequently, also include questions regarding the period for which the individual had access to improved standards of living.

## Figures and Tables

**Table 1:** Demographic characteristics of the study population.

Variables Indicated as *n* (%) or Median [IQR]	Normal CRP (<3 mg/L) *n* = 751	Elevated CRP (>3 mg/L) *n* = 818	*p*-Value ¤	Statistic ǂ	*p*-Value ∞
Men	317 (42.2%)	270 (33.0%)	<0.001	2.42 [0.72; 7.87]	<0.0002
Women	434 (57.8%)	548 (67.0%)		3.58 [1.24; 10.2]	
Menorrhea	229 (54.7%)	236 (43.6%)	0.001	3.05 [0.82; 9.00]	<0.0001
Amenorrhea	190 (45.3%)	305 (56.4%)		4.31 [1.72; 11.9]	
Age (years) *	46.0 [41.0; 53.0]	49.0 [42.0; 58.0]	<0.001	ρ = 0.12	<0.05
HIV-positive	128 (17.1)/	131 (16.1)/	*NS*	3.11 [0.93; 12.0]	*NS*
Negative	620 (82.9)	683 (83.9)		3.25 [0.96; 9.14]	
Tobacco use (whole group):					
Formerly	25 (3.4%)	33 (4.0%)	*NS*	3.98 [1.29; 18.3]	*NS*
Currently	403 (54.1%)	413 (50.6%)		3.05 [0.89; 9.18]	
Never	317 (42.6%)	370 (45.3%)		3.44 [1.04; 9.34]	
Alcohol consumption:					
Formerly	29 (3.9%)	38 (4.7%)	*NS*	3.74 [1.31; 14.1]	*NS*
Currently	321 (43.1%)	305 (37.6%)		2.78 [0.85; 9.28]	
Never	394 (53.0%)	469 (57.8%)		3.53 [1.05; 9.20]	
Body mass index (kg/m^2^)	21.6 [18.9; 24.9]	25.6 [19.7; 32.2]	<0.001	ρ = 0.24	<0.0001
Underweight	152 (20.4%)	134 (16.5%)		2.51 [0.64; 11.5]	
Healthy	407 (54.6%)	253 (31.1%)		1.89 [0.60; 5.67]	
Overweight	127 (17.0%)	145 (17.8%)		3.25 [1.30; 7.46]	
Obese	59 (7.9%)	282 (34.6%)		8.24 [3.74; 15.9]	
Waist circumference (cm) *	74.3 [68.5; 81.2]	82.4 [72.2; 92.9]	<0.001	ρ = 0.27	<0.0001
Hip circumference (cm) *	90.0 [83.8; 98.4]	98.2 [85.5; 112]	<0.001	ρ = 0.21	<0.0001
Dietary intake (kJ) *	6996 [5265; 9719]	7284 [5259; 10,025]	*NS*	ρ = 0.03	>0.05
Urban	388 (51.7%)/	420 (51.3)/	*NS*	3.20 [1.06; 9.83]	*NS*
Rural	363 (48.3%)	398 (48.7%)		3.26 [0.86; 8.75]	

* Continuous variables presented as median and interquartile (25th; 75th percentile]) ranges. ‡ Median CRP concentrations [interquartile ranges] calculated per grouping category or Spearman’s rho (ρ) for continuous variables. All other variables presented as the number of individuals (%) and median values. ¤ *p*-value for the difference in distribution of categorical variables of normal and elevated groups, or difference in continuous variables. ∞ *p*-values for difference in CRP concentrations within grouping variable between categorical variables or *p*-value for Spearman’s rho. Abbreviations: CRP C-reactive protein, HIV human immunodeficiency virus, IQR interquartile range (25th and 75th percentile), kJ kilojoule, *n* number of individuals, NS, not significant (*p* > 0.05).

**Table 2:** Factors of socio-economic status (SES) stratified according to baseline CRP cut-off values for elevated cardiovascular disease (CVD) risk.

Variables Indicated as *n* (%) or Median [IQR]		Normal CRP (<3 mg/L) *n* = 751	Elevated CRP (>3 mg/L) *n* = 818	*p*-Value ¤	Statistic ǂ	*p*-Value ∞
Education level	None	251 (34.1%)	286 (36.3%)	*NS*	3.33 [0.93; 9.91]	*NS*
	Primary	304 (41.4%)	334 (42.4%)		3.25 [1.01; 9.17]	
	Secondary	180 (24.5%)	167 (21.3%)		2.80 [0.83; 9.00]	
Marital status	Never married	282 (39.1%)	268 (33.5%)	0.04	2.86 [0.88; 8.69]	*NS*
	Partnered	357 (49.4%)	405 (50.7%)		3.36 [0.91; 9.34]	
	Separated	30 (4.2%)	45 (5.6%)		3.73 [1.21; 11.6]	
	Widowed	53 (7.34%)	81 (10.1%)		4.41 [1.65; 9.84]	
Time to nearest grocery store (minutes) *		30.0 [20.0; 60.0]	30.0 [20.0; 60.0]	*NS*	ρ = –0.015	*NS*
Time to nearest bank facility (minutes) *		30.0 [20.0; 60.0]	40.0 [20.0; 60.0]	*NS*	ρ = 0.018	*NS*
Access		639 (87.2%)	714 (88.8%)	*NS*	3.29 [0.97; 9.49]	*NS*
No access to electricity		94 (12.8%)	90 (11.2%)		2.91 [0.83; 7.76]	
Heat source	Coal open fire	92 (12.6%)	97 (12.2%)	*NS*	3.25 [0.97; 9.87]	*NS*
	Wood open fire	343 (47.1%)	342 (42.9%)		2.97 [0.84; 8.45]	
	Portable heater	28 (3.85%)	38 (4.76%)		4.17 [1.05; 15.3]	
	None	122 (16.8%)	129 (16.2%)		3.17 [0.92; 8.98]	
	Electricity	94 (12.9%)	131 (16.4%)		3.86 [1.42; 11.2]	
	Other	49 (6.7%)	61 (7.6%)		4.02 [1.25; 16.0]	
Water source	Sourced water	418 (57.1%)	440 (55.1%)	*NS*	3.25 [0.86; 9.05]	*NS*
	Municipal water	314 (42.9%)	359 (44.9%)		3.22 [1.13; 9.47]	
Roof structure	Galvanized iron sheets	601 (82.1%)	641 (79.7%)	*NS*	3.19 [0.90; 9.13]	*NS*
	Asbestos sheets	86 (11.7%)	112 (13.9%)		3.66 [1.21; 13.1]	
	Other	45 (6.2%)	51 (6.4%)		3.22 [1.10; 9.04]	
Cooking fuel	Electricity	275 (37.6%)	352 (43.8%)	*NS*	3.58 [0.99; 9.87]	*NS*
	Kerosene	224 (30.6%)	222 (27.6%)		2.96 [0.99; 9.28]	
	Gas	32 (4.4%)	35 (4.4%)		3.26 [1.05; 9.21]	
	Wood	188 (25.7%)	177 (22.0%)		2.87 [0.82; 8.86]	
	Other	45 (6.2%)	51 (6.4%)		3.22 [1.10; 9.04]	

* Continuous variables presented as median and interquartile (25th; 75th percentile) ranges. ǂ Median CRP concentrations (interquartile ranges) calculated per grouping category or Spearman’s rho (ρ) for continuous variables. All other variables presented as the number of individuals (%) and median values. ¤ *p*-value for the difference in the distribution of categorical variables of normal and elevated groups, or difference in continuous variables. ∞ *p*-values for the difference in CRP concentration within grouping variable between categorical variables or p-value for Spearman’s rho. Abbreviations: CRP C-reactive protein, IQR interquartile range (25th and 75th percentile), *n* number of individuals, NS not significant (*p* > 0.05).

**Table 3:** Physiological and biochemical markers of increased CVD risk.

Variables Indicated as *n* (%) or Median [IQR]	Normal CRP (<3 mg/L) *n* = 751	Elevated CRP (>3 mg/L) *n* = 818	*p*-Value ¤	Statistic ǂ	*p*-Value ∞
Systolic blood pressure (mmHg) *	127 [114; 144]	131 [117; 147]	0.002	ρ = 0.06	NS
Diastolic blood pressure (mmHg) *	85.0 [76.0; 94.0]	88.0 [79.0; 97.0]	<0.001	ρ = 0.08	NS
Heart rate (BPM) *	70.0 [61.0; 81.0]	73.0 [64.0; 87.0]	<0.001	ρ = 0.17	<0.0001
Hypertensive *	176 (23.6%)	228 (28.0%)	NS	3.63 [1.27; 8.88]	NS
normotensive *	569 (76.4%)	586 (72.0%)		3.11 [0.84; 9.32]	
Total cholesterol (mmol/L) *	4.76 [4.02; 5.79]	4.95 [4.03; 6.01]	0.035	ρ = 0.04	>0.05
HDL-c (mmol/L) *	1.48 [1.14; 1.98]	1.34 [1.02; 1.80]	<0.001	ρ = −0.15	<0.0001
LDL-c (mmol/L) *	3.01 [2.32; 3.77]	3.23 [2.44; 4.14]	<0.001	ρ = 0.1	<0.0001
Triglycerides (mmol/L) *	1.01 [0.76; 1.41]	1.14 [0.85; 1.65]	<0.001	ρ = 0.144	<0.0001
HbA1c (%) *	5.40 [5.20; 5.70]	5.60 [5.30; 5.90]	<0.001	ρ = 0.23	<0.0001

* Continuous variables presented as median and interquartile [25th; 75th percentile] ranges. ǂ Median CRP concentrations [interquartile ranges] calculated per grouping category or Spearman’s rho (ρ) for continuous variables. All other variables presented as the number of individuals (%) and median values. ¤ *p*-value for the difference in the distribution of categorical variables of normal and elevated groups, or difference in continuous variables. ∞ *p*-values for the difference in CRP concentration within grouping variable between categorical variables or p-value for Spearman’s rho. Abbreviations: BPM beats per minute, CRP C-reactive protein, HbA1c glycated hemoglobin, HDL-c high-density lipoprotein cholesterol, IQR interquartile range (25th and 75th percentile), kJ kilojoule, LDL-c low-density lipoprotein cholesterol, mmHg millimeters of mercury, *n* number of individuals, NS not significant (*p* > 0.05).

**Table 4:** Natural log-transformed CRP concentrations as a function of covariates.

Variable	Estimate β Coefficients	Standard Error	Change (%) *	*p*-Value
Intercept	−3.387	0.388		<0.0001
Age	0.014	0.004	1.41	0.0002
Heart rate	0.020	0.002	2.21	<0.0001
WC	0.027	0.003	3.10	<0.0001
HDL-C	−0.249	0.059	−22.0	<0.0001
HbA1c	0.117	0.043	12.4	0.006
Completed at least seven years of formal education	−0.090	0.085	−8.60	0.292
Twelve or more years of formal education	−0.209	0.104	−18.9	0.044

* % change in CRP calculated for a 1 unit change in covariate [(e^β^ − 1) × 100)]. Abbreviations: HbA1c glycated hemoglobin, HDL-c high-density lipoprotein cholesterol, WC waist circumference.

## Supplementary Materials

The following are available online at https://www.mdpi.com/1660-4601/17/18/6646/s1, Table S1: CRP SNPs, their minor allele frequencies.
