# Supplementary material for: Education, Smoking and CRP Genetics in Relation to C-Reactive Protein Concentrations in Black South Africans
Source: Int J Environ Res Public Health. 2020 Sep 11;17(18):6646. doi: 10.3390/ijerph17186646 (PMC7558133; doi:10.3390/ijerph17186646)
Supplement: Supplementary file 1 [file ijerph-17-06646-s001.pdf]

Supplemental Table 1: *CRP* SNPs, their minor allele frequencies.

rs number (SNP location)	SNP pseudonyms	Genotype count (genotype frequency %)		95% CI of genotype frequency (%)	MAF (%) [1000 Genomes Project MAF% for Africans / Europeans]	HW p-value
rs7553007[Φ]<br>(159728759)	Promotor	GG<br>GA<br>AA	923 (57.90)<br>583 (36.57)<br>88 (5.52)	55.4 – 60.3<br>34.2 – 39.0<br>4.43 – 6.70	A = 23.8<br>[19.8 / 30.9]	0.05
rs1341665[Φ]<br>(159721769)	-7180C>T<br>Promotor	GG<br>GA<br>AA	931 (58.44)<br>577 (36.22)<br>85 (5.34)	55.9 – 60.8<br>33.9 – 38.6<br>4.27 – 6.49	A = 23.5<br>[19.0 / 31.6]	0.53
rs2027471[Φ]<br>(159719598)	Promotor	TT<br>TA<br>AA	932 (58.47)<br>577 (36.20)<br>85 (5.33)	55.9 – 60.8<br>33.9 – 38.6<br>4.26 – 6.49	A = 23.5<br>[18.8 / 31.7]	0.73
rs3093058[§]<br>(159715525)	-790A>T<br>Promotor (conserved region predicted to alter a transcription factor E box binding element)	AA<br>AT<br>TT	1101 (69.25)<br>458 (28.81)<br>31 (1.95)	67.1 – 71.6<br>26.5 – 31.0<br>1.23 – 2.58	A = 19.4 [18.4 / 0.0]	0.84
rs3093062[§]<br>(159714894)	-409G>A<br>Promotor	GG (CC)<br>GA (CT)<br>AA (TT)	1102 (69.26)<br>458 (28.79)<br>31 (1.95)	67.1 – 71.7<br>26.5 – 30.9<br>1.23 – 2.58	T = 16.4 [18.5 / 0.0]	0.76
rs1417938<br>(159714396)	IVS1+29A>T, i178T>A; 1919A>T<br>intron (position + 29) close to splice site junction	AA<br>TA	1509 (95.03)<br>79 (4.97)	94.0 – 96.2<br>3.82 – 5.96	A = 2.49 [8.50 / 32.6]	0.27
rs1800947<br>(159713648)	Leu184Leu (L184L) or silent/synonymous mutation or +1059C>G; 2667G>C<br>Exon 2	CC<br>CG	1587 (99.56)<br>7 (0.44)	99.2 – 99.9<br>0.12 – 0.77	G = 0.22 [0.20 / 0.51]	0.62
rs1130864<br>(159713301)	3u1273C>T or +1444G>A or 3014G>A<br>3′ UTR	CC (GG)<br>CT (GA)<br>TT (AA)	1189 (74.59)<br>382 (23.96)<br>23 (1.44)	72.4 – 76.7<br>21.9 – 26.1<br>0.86 – 2.05	A = 13.4 [13.2 / 32.7]	0.05
rs1205[Φ]<br>(159712443)	3u2131C>T or 3872A>G/C>T or +1846G>A<br>Distal 3′ UTR (′3 flanking region) in the MLT1K repeat	CC<br>CT<br>TT	945 (59.32)<br>570 (35.78)<br>78 (4.90)	56.9 – 61.7<br>33.4 – 38.2<br>3.81 – 5.94	T = 22.8 [17.0 / 31.0]	0.73
rs3093068<br>(159711574)	+2911C>G; 4741G>C<br>3′ UTR	GG<br>GC<br>CC	610 (38.32)<br>769 (48.30)<br>213 (13.38)	35.9 – 40.7<br>46.1 – 51.0<br>11.5 – 14.8	C = 37.5 [29.9 / 7.10]	0.29
rs2808630<br>(159711078)	+5237A>G<br>3′ UTR	TT<br>TC<br>CC	1173 (73.59)<br>387 (24.28)<br>34 (2.13)	71.4 – 75.7<br>22.2 – 26.4<br>1.43 – 2.87	C = 14.3 [14.8 / 28.4]	0.73
rs2794520[Φ]<br>(159709026)	3′ UTR	CC<br>CT<br>TT	923(58.27)<br>574 (36.24<br>87 (5.49)	55.8 – 60.7<br>33.9 – 38.7<br>4.34 – 6.60	T = 23.6 [18.3 / 31.1]	1.00

Several SNP aliases are the complement alleles of those given on Ensembl or published elsewhere. At rs3093058 we report our raw BeadXpress data even though it differs from that of Ensembl and previously reported by our group. Major/minor allele frequency is based Homo Sapiens GRCh38.p5.
